# Cytomegalovirus-Specific T Cells from Third-Party Donors Successfully Treated Refractory Cytomegalovirus Retinitis after Unrelated Umbilical Cord Blood Transplantation

**DOI:** 10.1155/2022/6285510

**Published:** 2022-11-15

**Authors:** Na Li, Guangyu Sun, Lihua Zhu, Kang Ding, Huilan Liu, Xiaoyu Zhu, Baolin Tang, Wen Yao, Xiang Wan, Liangquan Geng, Ping Qiang, Kaidi Song, Changcheng Zheng, Zimin Sun, Juan Tong

**Affiliations:** ^1^Wannan Medical College, Graduate School, Anhui Wuhu 241002, China; ^2^Department of Hematology of Anhui Provincial Hospital, The First Affiliated Hospital of University of Science and Technology of China, Hefei, China

## Abstract

Umbilical cord blood (UCB) transplants (UCBTs) are becoming increasingly common in the treatment of a variety of hematologic and nonhematologic conditions. The T cells from UCB are naïve T cells, which have not yet been exposed to antigens and therefore do not contain T cells with specific immune functions against viruses. Cytomegalovirus (CMV) infections occur in more than 80% of patients after UCBT compared to other types of transplantation. Anti-CMV medications are currently restricted, with ganciclovir, foscarnet, and valganciclovir being the most common in China; however, with limited efficacy and considerable side effects, all these drugs are susceptible to viral resistance. In recent years, cytomegalovirus-specific T cells (CMVST) have advanced the treatment of viral infections in immunodeficient patients. CMVST usually uses the same donor as hematopoietic stem cell transplantation. CMVST should be administered to UCBT patients because of the absence of donors after UCBT. In China, there is no report on the use of CMVST to treat CMV infection after UCBT, and foreign reports are also limited. This paper reported a 20-year-old male patient with acute myeloid leukemia who developed cytomegalovirus retinitis (CMVR) after umbilical cord blood transplantation. After ineffective viral treatment, he was treated with a third-party donor CMVST and was successfully transformed into CMV nucleic acid negative.

## 1. Introduction

Since the first report in 1989, umbilical cord blood (UCB) stem cells have been successfully used in hematopoietic cell transplantation (HCT) [1]. Since then, more than 40000 UCB transplants have been performed worldwide for a variety of malignant and nonmalignant diseases [2, 3]. In hematological malignancies, umbilical cord blood transplantation (UCBT) is as effective as related or unrelated bone marrow or peripheral blood as the source of transplantation [4, 5]. One of the most common side effects of UCBT is cytomegalovirus (CMV) infection, which can result in pneumonia, enteritis, encephalitis, retinitis, and other complications, with a very high mortality and disability rate that significantly affects the success of UCBT and the life quality of patients [6]. There are currently only a few types of anti-CMV medicines available, with ganciclovir (GCV) and foscarnet serving as first-line antiviral therapies [7]. These drugs can inhibit the reconstruction of cytomegalovirus-specific T cells (CMVST), leading to an increased possibility of developing advanced CMV infection (infection that happens during the first 100 days after transplantation), a condition that is associated with substantial target organ damage, a high rate of medication resistance, and a high mortality rate, all of which lead to increasingly serious consequences.

The use of CMVST to treat infections in immunocompromised people has recently received considerable interest. The progress in recent years has also been very rapid, developing from targeting a single virus to the current ability to target multiple viruses simultaneously and from the initial need to harvest blood from a hematopoietic stem cell donor to now being able to harvest blood from a third-party healthy donor, offering new hope for the treatment of those without a donor after UCBT [8]. Tzannou et al. [9] reported that a third-party donor source could be used for simultaneous treatment of five viruses, including CMV, EBV, ADV, BKV, and HHV-6. At present, developed countries in Europe and the United States have begun to establish virus-specific T cell (VST) libraries from third-party donors. In the United States, for example, more than 500 cells are stored in a cell bank where most virus-infected patients can quickly find the appropriate VST. Such a VST bank has not yet been established in China, but VST donors can be found in the parents, children, siblings, and other HLA semi-identical immediate relatives of UCBT patients. The preparation process of CMVST has also improved significantly, allowing it to be used for treatment 2-3 weeks after blood collection. In recent years, countries have reported antiviral efficiency rates as high as 80%-100% [10]. Antiviral drug therapy combined with CMVST treatment significantly reduces the risk of CMV infection and reduces persistent CMV infection [11]. CMV infection in other immunodeficiency patients (such as cancer, AIDS, and organ transplantation) can also be treated with CMVST.

## 2. Case Data

### 2.1. Basic Information and Diagnosis

The patient was a 20-year-old male college student from Anhui Province who was admitted to the Department of Hematology of the Affiliated Hospital of the University of Science and Technology of China on February 7, 2019, with a fever for 2 days. After admission, he completed relevant examinations. On February 7, the routine blood test showed white blood cells 44.69 × 10^9^/L, red blood cells 1.13 × 10^9^/L, hemoglobin 41.0 g/L, platelets 47 × 10^9^/L, and neutrophils 5.1 × 10^9^/L. Blast cells accounted for 82% of the peripheral blood classification on February 8 and 83.5% on February 11 by the bone marrow cytology morphology, with myelogram indicating partially differentiated acute granulocytic leukemia (AML-M2a). Immunophenotyping on February 14 showed that acute myeloid leukemia with partial expression of CD19 was possible. On February 19, chromosomal analysis revealed normal 46, XY karyotype, and negative AML prognostic genes. The final diagnosis was acute myeloid leukemia (M2a, normal chromosome, intermediate-risk group).

### 2.2. Treatment History

After the patient was diagnosed with AML (M2a, normal chromosomes, intermediate-risk group) in February 2019, he was given an IA regimen (idarubicin at 12 mg/m^2^ d1-d3, cytarabine at 100 mg/m^2^ d1-d7) on February 10, 2019. On March 18, he was admitted for evaluation of his condition, and the minimal residual disease (MRD) test reported 9.4% aberrant myeloid blast cells in the posttreatment material. On March 19, he was administered a FLAG regimen (fludarabine at 30 mg/m^2^ d1-5, cytarabine at 2.0 g/m^2^ d1-5, granulocyte colony-stimulating factor at 450 *μ*g qd) to trigger chemotherapy again after a previous course failed to achieve complete remission (CR).

On April 11, the myelogram was reperformed, and the number of myeloblasts was found to be approximately 2.0%. On April 16, the MRD test report indicated that 0.53% of abnormal myeloid immature granulocytes were found within the scope of this test after treatment. It was considered to achieve bone marrow morphological remission (BMR). The patient was administered consolidation chemotherapy consisting of decitabine and high-dose cytarabine (decitabine at 20 mg/m^2^ d1-d5, cytarabine at 1.5 g/m^2^ q12h, d3-d5) on April 20. Then, he was treated with unrelated UCBT after the myeloablative conditioning regimen from June 5 with FLU+BU+CY+BCUN (30 mg/m^2^ fludarabine from d6 to d3, 0.8 mg/kg busulfan from d5 to d2, 60 mg/kg cyclophosphamide from d2 to d0, and 250 mg/m^2^ carmustine on d6). A series of treatments were also given to prevent veno-occlusive disease (VOD) and to provide liver and stomach protection and infection prevention.

Under cardiac monitoring, the patient received 23 ml of UCB stem cells (HLA5/6, 8/10, A+ to A+, male to male) from the Beijing bank on June 15. The total counts of nucleated cells, CD34^+^ cells, CD^+^ cells, and CD56^+^ cells were 3.33 × 10^7^/kg, 2.36 × 10^5^/kg, 2.51 × 10^6^/kg, and 3.51 × 10^6^/kg, respectively. Short tandem repeat-polymerase chain reaction (STR-PCR) detection performed on the 14th day after transplantation showed 100% donor chimerism. Neutrophil engraftment occurred on day +18 and platelet engraftment on day +27. After transplantation, the patient received cyclosporine A (CSA) and mycophenolate mofetil (MMF) for graft versus host disease (GVHD) prophylaxis. On July 8, the patient showed an increased number of stools, and the stool routine showed positive occult blood. Combined with fecal flora analysis, GVHD was considered. The patient was given mycophenolate mofetil, ruxolitinib, CD25 monoclonal antibody (CD25 mAb), and methotrexate to treat GVHD.

On the second day after transplantation, the patient was started on acyclovir (0.3 g q12 h) for prophylaxis of CMV infection. However, on July 12 (on the 27th day after transplantation), the CMV nucleic acid test (PCR) was positive (3.16E+2), confirming CMV infection. The patient started to use the first-line antiviral drug ganciclovir (250 mg q12 h) intravenously on July 29 (on the 44th day after transplantation) because the CMV nucleic acid test was still positive several times during the period (the maximum number of copies was 9.49E+2); however, the virus copy number did not decrease (the maximum number of copies was 1.02E+4), indicating ineffective antiviral drug treatment. On September 17 (on the 94th day after transplantation), the patient's CMV nucleic acid detection (PCR) value was higher than previously reported (4.37E+2). Ganciclovir was stopped, and foscarnet (3 g q12 h) was substituted until September 26, 2019. On September 21, the patient had abnormal urination. Urine routine tests showed urinary red blood cells (KB) > 71.3/*μ*l, bacteria (KB) 218.5/*μ*l, protein ++, occult blood (or) red blood cells ++, and positive CMV-PCR in the whole blood and urine (7.18E+2 and 5.78E+2, respectively), suggesting hemorrhagic cystitis (HC). The patient continued to receive antiviral, immunomodulatory, and anti-GVHD support treatments; HC gradually improved, and CMV and blood in urine were negative three months later.

On July 30, 2020, the patient went to the Department of Ophthalmology of Xinhua Hospital Affiliated with the Medical College of Shanghai Jiaotong University School of Medicine due to blurred binocular vision in both eyes for more than 4 months. The auxiliary examination showed VOD: 0.05, VOS: 0.05, NCT: 15.6 mmHg(od), and 15.7 mmHg(os); the cornea of both eyes and the anterior chamber was clear, the pupil was round, and the direct and indirect light response was good, with mixed posterior capsule and the retina of both eyes showing multiple yellow and white lesions; the preliminary diagnosis was cytomegalovirus retinitis (CMVR). On August 6, ganciclovir (2.5 mg/0.1 ml) was injected into the vitreous cavity of both eyes. On August 14, the patient was diagnosed with CMV infection in the aqueous humor, with 6.33E+4 in the right eye and 3.43E+4 in the left eye. From July 30 to September 21, ganciclovir was infused into the vitreous cavity of both eyes 6 times. After the fifth infusion, CMV in the aqueous humor was still positive, with 1.34E+4 in the right eye and 8.68E+3 in the left eye. CMV in the aqueous humor did not turn negative after the 6th infusion. On September 27, the patient went to the Hematology Department of the Affiliated Hospital of the University of Science and Technology of China due to fever and granulocyte deficiency. Granulocyte deficiency is considered a side effect of GCV [12]. After the treatment of stimulating hematopoiesis and anti-infection, the granulocytes and the body temperature all returned to normal. In October, the Hematology Department of the Affiliated Hospital of the University of Science and Technology of China evaluated the patient before CMVST transfusion, favoring CMVST transfusion.

Peripheral blood (80 ml) from a third-party hemiphasic donor (patient's brother, HLA 5/10 compatible) was drawn, and the individual nucleated cells were purified and placed in highly permeable G-REX culture flasks for culture. Microbeads loaded with CD3 and CD28 antibodies were added to polyclonally activate the T cells, followed by the addition of 1000 U/ml IFN-*γ* on day 0, as well as 500 U/ml IL-2 and 100 ng/ml anti-CD3 antibody 24 hours later. High purity (approximately 90%) CD3 T cells were obtained after 7 days of culture, and the T cells generated by this step of expansion were polyclonal T cells, of which memory T cells (TM) accounted for more than 90%. After the collection of polyclonal T cells, pp65 antigen peptides of CMV (purchased from Molten, Germany) were added. A total of 138 peptides were added, each with a length of 15 aa and 10 aa sequence identity between adjacent peptides, covering the entire protein sequence. When these antigenic peptides were incubated with TM, at least one of them was embedded in the HLA molecule on the cell surface and thus specifically recognized by the TCR on the CMVST surface. The proliferation capacity of such antigen-specific activated CMVST is approximately 10 times greater than that of inactivated TM. After approximately 20 days of differential amplification, the percentage of CMVST usually reaches 20% or more. After 7 days of culture, quality control tests were performed, and cells that met the release criteria were collected on day 10 of culture, of which approximately 50% were used for the first treatment; the other 50% were restimulated by the pp65 antigen peptide fragment and continued to be cultured for 10 days for the second treatment.

On October 23, 200 ml of specific T cells was infused for the first time under cardiac monitoring (the number of cells was 8.92 × 10^9^, and the number of specific T cells was 1.96 × 10^9^, calculated as the form of cell viability per kilogram of body weight, 97%). The process went smoothly without special discomfort, and the patient showed no fever, rash, hypotension, or anaphylactic shock within 2 hours of infusion. On October 23, the peripheral blood CMV nucleic acid test (PCR) was positive (CMV-DNA). On November 3, 2020, 200 ml of specific T cells was infused for the second time under cardiac monitoring (the number of cells was 8.92 × 10^9^, and the number of specific T cells was 1.96 × 10^9^, calculated as the form of cell viability per kilogram of body weight, 97%). The process was smooth without cytokine release syndrome (CRS) or GVHD. On November 3, the peripheral blood CMV nucleic acid detection (PCR) showed negative. And the CMV nucleic acid test (PCR) of the aqueous humor was negative after the second CMVST infusion. At follow-up on April 1^st^, 2022, the patient was negative for CMV nucleic acid after CMVST infusion and had no relapse. The CMV nucleic acid test in the aqueous humor and peripheral blood was also negative on April 1^st^, 2022.

**Figure 1:**
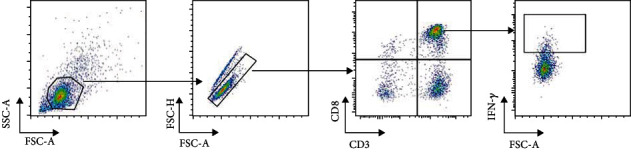
Cell shooting of flow cytometry.

**Figure 2:**
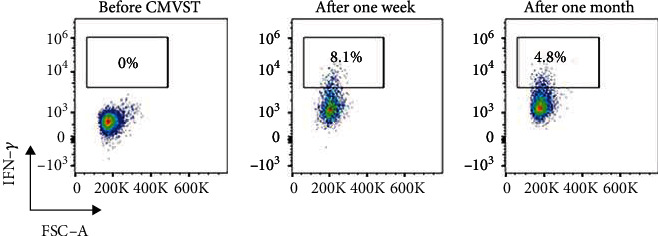
IFN-*γ*^+^CD_8_^+^ cells before and after CMVST infusion.

Before CMVST infusion, the T cell subsets showed CD3^+^ at 74.5%, CD3^+^ CD4^+^ at 25%, CD3^+^ CD8^+^ at 46.9%, and CD3^+^ CD4^+^/CD3^+^ CD8^+^ at 0.53%. One week after the second infusion, the T cell subsets showed CD3^+^ at 74.6%, CD3^+^ CD4^+^ at 20.8%, CD3^+^ CD8^+^ at 50.6%, and CD3^+^ CD4^+^/CD3^+^ CD8^+^ at 0.41%. One month after the second infusion, the T cell subsets showed CD3^+^ at 73.6%, CD3^+^ CD4^+^ at 18.3%, CD3^+^ CD8^+^ at 51.6%, and CD3^+^ CD4^+^/CD3^+^CD8^+^ at 0.36%. It is suggested that the number of CD8^+^ T cells increased slightly after infusion and remained stable for one month after infusion (Table 1). We also analyzed the proportion of IFN-*γ*^+^CD8^+^ cells (Rapid Cytokine Inspector CD4/CD8 T-cell kit; Miltenyi Biotec) before CMVST infusion as well as one week and one month after the second CMVST infusion. The proportion of IFN-*γ*^+^CD8^+^ cells was 0% before CMVST infusion and 8.1% and 4.8% at one week and one month after the second CMVST infusion, respectively (Figures 1 and 2).

## 3. Discussion

CMVR is commonly seen in immunocompromised patients. Because of their lowered immunity after UCBT, patients are extremely vulnerable to pathogenic infections [13]. CMVR is a common opportunistic infection in patients undergoing allogeneic hematopoietic stem cell transplantation (allo-HSCT). A diagnosis is difficult to make due to nonspecific early clinical symptoms of CMVR, and if effective treatment is not provided early, it can progress to retinal necrosis, retinal detachment, optic nerve atrophy, and even blindness [14].

Ganciclovir, foscarnet, cidofovir, and valganciclovir are currently the most common CMVR treatment medications in China. These antiviral medications work by inhibiting viral reproduction, but they do not eliminate the virus altogether and are associated with a variety of side effects [15–17], inducing bone marrow suppression, nephrotoxicity, and gastrointestinal reactions. Therefore, markers of routine blood, liver function, and renal function must be continuously monitored in individuals who have become immunocompromised following UCBT [18–23]. Intralesional ganciclovir injection can lead to decreased visual acuity, increased intraocular pressure, and even damage to the optic nerve and retina as well as infection [24, 25]. Antiviral medicines implanted intravitreally induced endophthalmitis, retinal detachment, and other adverse events, according to two retrospective studies [26, 27]. Therefore, VST-mediated immunity plays a crucial role in anti-CMV treatment. The excellent efficacy of CMVST infusion for CMVR treatment has been demonstrated in the research by Gupta et al. [28]. A 26-year-old male patient with a history of acute lymphoblastic leukemia developed CMV viremia and esophagitis sequentially after allogeneic transplantation [29]. He received antiviral drugs, such as valganciclovir and foscarnet, but without improvement. Later, he developed CMVR and retinal OD deteriorated after binocular vitreous injections of ganciclovir (2 mg/0.1 ml) showing ineffective. After treatment with CMV pp65-specific cytotoxic T cells (CMVpp65 CTLs) from a third-party donor source, retinitis completely resolved and remained inactive during the 3-month follow-up.

China now mostly employs antiviral drugs to treat CMV infection following transplantation; however, there are still several flaws. CMVST, in comparison to the primary domestic antiviral medications, eliminates CMV through immunotherapy, with precise curative effects demonstrated and no side effects. Acyclovir (0.3 g q12 h) was initiated for prophylaxis of CMV infection on the second day after transplantation in this case. The patient tested positive for CMV nucleic acid on the 32nd day after transplantation and successively developed intestinal infection, pulmonary infection, HC, and CMVR. The patient's CMV nucleic acid test showed no negative conversion after therapy with antiviral medicines, such as ganciclovir and foscarnet. The patient's CMV nucleic acid test came back negative after the second CMVST infusion on November 3, 2020. The patient did not experience acute or chronic GVHD during follow-up, and the peripheral blood and atrial fluid CMV nucleic acid testing was negative. Pei et al. revealed that adoptive therapy with CMVST is a safe and effective treatment for CMV infection after haploidentical stem cell transplantation. Refractory CMV infection and basiliximab treatment are correlated with poor anti-CMV efficacy of CMV-CTL therapy [30].

The risk of GVHD, the most common side effect of CMVST, can be reduced by controlling the number and purity of CMVST cells. Mild acute GVHD has been described in a few patients after CMVST infusion [31], but the incidence is less than 1% and was not necessarily related to CMVST infusion. CMVST has the advantages of precise efficacy, few side effects, and low resistance rate, while not interfering with normal CMVST reconstruction in patients, which can reduce the risk of advanced CMV infection.

## 4. Conclusion

CMVST from a third-party hemiphasic donor appears to be a viable treatment for refractory CMV infection after UCBT based on this patient's successful treatment of CMVR. Hopefully, CMVST from a third-party hemiphasic donor can be promoted in clinical treatment of CMV infection.

## Figures and Tables

**Table 1:** T cell subsets after CMVST infusion.

	CD3^+^	CD3^+^ CD4^+^	CD3^+^ CD8^+^	CD4^+^/CD8^+^
Before CMVST infusion	74.5%	25%	46.9%	0.53%
One week after the second time of infusion	74.6%	20.8%	50.6%	0.41%
One month after the second time of infusion	73.6%	18.3%	51.6%	0.36%

Note: the first infusion of CMVST was given on October 23, 2020, and the second infusion of CMVST was given on November 3, 2020.

## Data Availability

The labeled dataset used to support the findings of this study are available from the corresponding author upon request.
